# Supplementary figures and images for: Treatment of Carbapenem-Resistant Acinetobacter baumannii in Real Life (T-ACI): A Prospective Single-Center Observational Study
Source: Antibiotics (Basel). 2024 Oct 25;13(11):1007. doi: 10.3390/antibiotics13111007 (PMC11591046; doi:10.3390/antibiotics13111007)

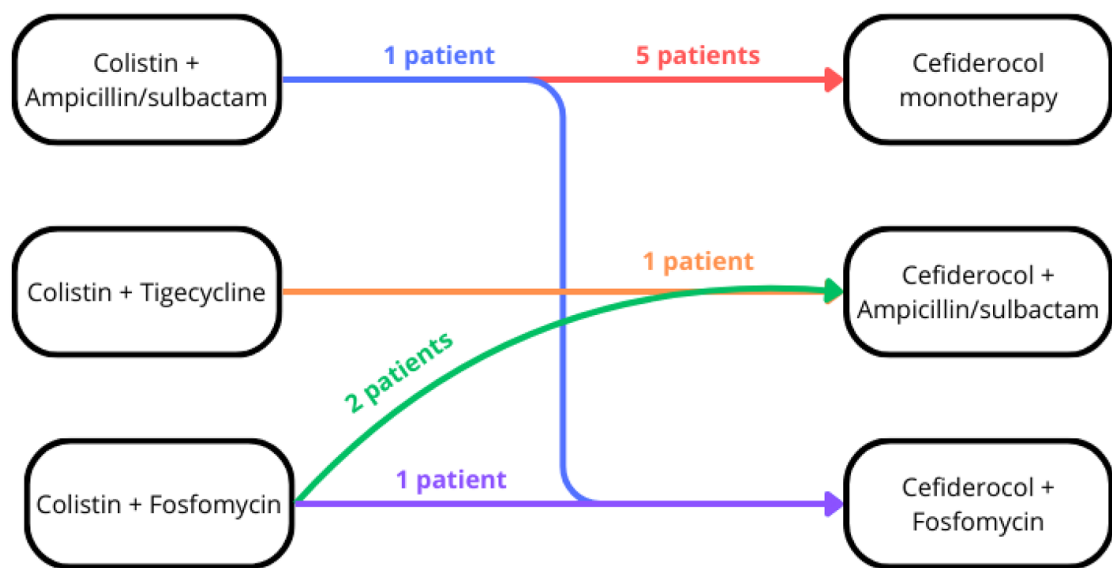

**Figure S1.** Antibiotic switches.

Supplement: Supplementary file 1 [file antibiotics-13-01007-s001.zip › antibiotics-3255250-supplementary.pdf]
